# Criegee intermediate-hydrogen sulfide chemistry at the air/water interface†
†Electronic supplementary information (ESI) available: Radial distribution functions for the H_2_S–H_2_O and CH_2_OO–H_2_O interactions; probability distributions *P*(*θ*_1_,*θ*_2_) for H_2_S at the air–water interface; videos of trajectories of the BOMD simulations for the concerted and stepwise reactions between CH_2_OO and H_2_S at the air/water interface; BLYP/aug-cc-pVTZ-optimized geometries of key stationary points for the gas-phase reactions of CH_2_OO with H_2_S and H_2_S–H_2_O, respectively. See DOI: 10.1039/c7sc01797a



**DOI:** 10.1039/c7sc01797a

**Published:** 2017-05-16

**Authors:** Manoj Kumar, Jie Zhong, Joseph S. Francisco, Xiao C. Zeng

**Affiliations:** a Department of Chemistry , University of Nebraska-Lincoln , Lincoln , Nebraska 68588 , USA . Email: xzeng1@unl.edu ; Email: jfrancisco3@unl.edu; b Beijing Advanced Innovation Center for Soft Matter Science and Engineering , Beijing University of Chemical Technology , Beijing 100029 , China

## Abstract

We carry out Born–Oppenheimer molecular dynamic simulations to show that the reaction between the smallest Criegee intermediate, CH_2_OO, and hydrogen sulfide (H_2_S) at the air/water interface can be observed within few picoseconds. The reaction follows both concerted and stepwise mechanisms with former being the dominant reaction pathway. The concerted reaction proceeds with or without the involvement of one or two nearby water molecules. An important implication of the simulation results is that the Criegee-H_2_S reaction can provide a novel non-photochemical pathway for the formation of a C–S linkage in clouds and could be a new oxidation pathway for H_2_S in terrestrial, geothermal and volcanic regions.

## Abstract 2

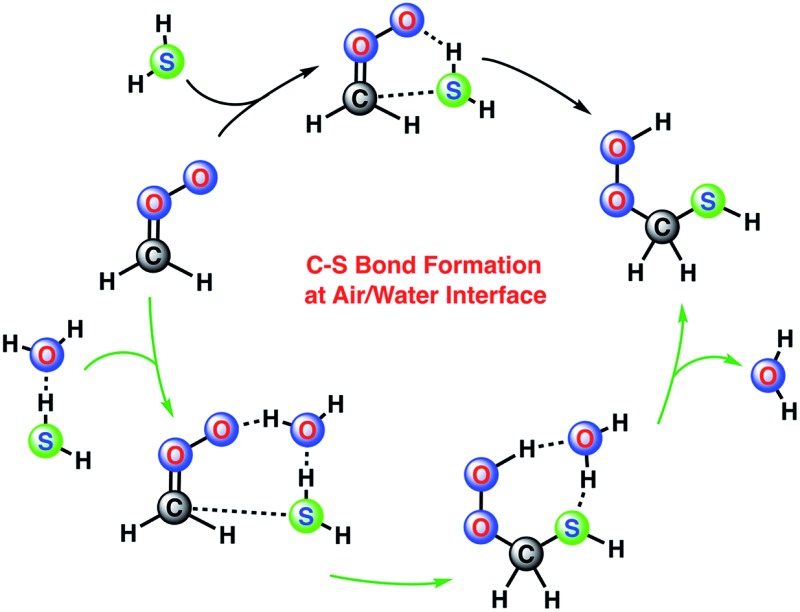
We carry out Born–Oppenheimer molecular dynamic simulations to show that the reaction between the smallest Criegee intermediate, CH_2_OO, and hydrogen sulfide (H_2_S) at the air/water interface can be observed within few picoseconds.

## 

Carbon–sulfur linkages are ubiquitous in atmospheric, combustion, and biological chemistries. Thioaldehydes comprise an important class of carbon–sulfur compounds that has a broad chemical appeal. For example, thioformaldehyde (HCHS) has been detected in dark clouds, the interstellar clouds^1^ and circumstellar envelope around an asymptotic giant branch star.^2^ HCHS is also believed to play a key role in the photochemical evolution of sulfur-containing species in the Earth's atmosphere and other astronomical systems.^1c,2^ In synthetic organic chemistry, thioaldehydes are used as key building blocks.^3^ The high intrinsic reactivity and polarisability of thioformyl group makes possible the construction of new carbon bonds with remarkable selectivity.^4^ Thioaldehydes also possess potential biological activities.^5^ Recently, thioaldehydes have been implicated as common intermediates in biosynthetic pathways in methanogens.^6,7^ Despite their broad profile, the laboratory synthesis of simpler aliphatic thioaldehydes has been a great challenge because of their tendency towards oligomerization.^8^

In the atmosphere, thioaldehydes are believed to be formed by the nucleophilic attack of SH^–^ ion on the carbonyl group. However, it has recently been suggested that the gas-phase reaction between Criegee intermediate or a carbonyl compound and hydrogen sulfide (H_2_S) under water or acid catalysis could account for the atmospheric formation of thioaldehydes and provide useful guidelines for efficiently synthesizing thioaldehydes within laboratory environment.^7,9^ Criegee intermediates are formed in the cycloaddition reactions between ozone and olefins.^10^ Ever since Criegee intermediates have directly been detected in the gas phase,^11^ understanding their chemistry using experimental and theoretical means has become an important avenue of atmospheric research.^12^ Criegee intermediates are not only important in atmospheric context, but also play a key role in enzymatic reactions,^13^ pharmaceutical pathways and general synthetic organic reactions.^14^ H_2_S is released into the air naturally and as a result of human activity. Natural sources, such as swamps, bogs, natural gases, geothermal hot springs, fumaroles and volcanoes, account for about 90% of the total amount of H_2_S in the atmosphere.^15^ Typical H_2_S concentrations in volcanic and geothermal regions are about 500 parts per billion.^16^ However, gypsum drywells present in the construction and demolition wastes could produce significant amounts of H_2_S ranging from 7 parts per million (ppm) to 100 ppm.^17^ In certain cases, more dangerous levels of 5000–12 000 ppm were also measured.^18^ H_2_S is also emitted by plants.^19^ Estimates of the terrestrial emission rates of H_2_S range from 58 to 110 million tons of sulfur per year.^20^ There is significant literature suggesting that in the forested environments,^21^ the Criegee intermediates could be formed from the interaction of terpenes with ozone. Clearly, the thioaldehyde-forming Criegee-H_2_S chemistry could be locally important in the troposphere.

Aerosols, fog and clouds are believed to play a key role in atmospheric chemistry.^22^ In the atmosphere, the abundance of aerosols can rise up to ∼10^8^ to 10^9^ m^–3^, and the maximum surface area of the aerosols in clouds can be ∼10^–9^ m^2^.^23^ This suggests that the air/water interface, which is characteristic of the surface of oceans, lakes, and atmospheric aerosols, may also play a more direct role in the Criegee reactions due to their ability to concentrate and align reacting species in a water restricting environment. However, most of the Criegee reactions have only been examined in the gas phase until recently^22*b*^ when the dynamics of the Criegee-water (H_2_O) reaction at the air/water interface was investigated in detail using the adaptive buffered force Quantum Mechanics/Molecular Mechanics dynamics simulations (adbf-QM/MM). Contrary to the previously established concerted mechanism in the gas-phase, a significant fraction of the Criegee-H_2_O reaction at the air/water interface follows a stepwise mechanism.

In the atmosphere, the major sink of H_2_S is assumed to be its reaction with the OH radical. However, nearly half of H_2_S emitted into the troposphere comes from ocean,^24^ suggesting that the aqueous surface chemistry of H_2_S could play an important role in its tropospheric oxidation under certain conditions. In this article, we show a direct evidence, based on the Born–Oppenheimer molecular dynamics (BOMD) simulations, that the reaction between the simplest Criegee intermediate, CH_2_OO, and H_2_S at the air/water interface occurs within few picoseconds and results in the formation of thioaldehyde. In the terrestrial regions, the Criegee intermediate and H_2_S may first react in the gas-phase to form the Criegee-H_2_S complex, which then could get adsorbed on the water droplet and allow the subsequent reaction. The reaction at the air–water interface follows both concerted and stepwise mechanisms. To our knowledge, this is the first evidence that suggests the role of the water droplets (*e.g.*, clouds) in catalysing the non-photochemical formation of a C–S linkage. These results not only reveal a general effect of the water droplet on the Criegee-H_2_S chemistry, but also offer valuable insights into the tropospheric oxidation of H_2_S on the aqueous surface.

## Methods

**Fig. 1:**
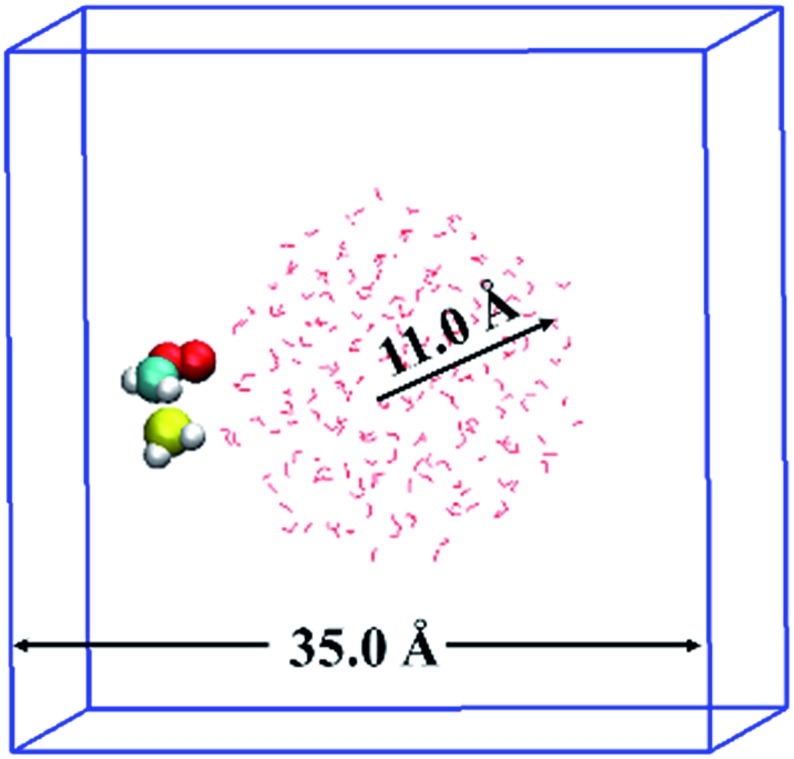
Initial configuration of Criegee intermediate, CH_2_OO and hydrogen sulphide, H_2_S adsorbed on the water droplet of 191 water molecules. The white, red, cyan and yellow colours represent H, O, C and S atoms, respectively. CH_2_OO and H_2_S are shown in ball–stick representation whereas the water droplet is shown in line representation.

The BOMD simulations were performed on the basis of density functional theory (DFT) method implemented in the CP2K^25^ code. The reaction between CH_2_OO and H_2_S at the air/water interface is investigated using a model system that contained 191 H_2_O molecules, one CH_2_OO molecule, and one H_2_S molecule, as shown in Fig. 1. The solvation structure of H_2_S and CH_2_OO adsorbed on the water droplet was also examined. The radius of the water droplet in our system is about 11 Å. The dimensions of the simulation box are *x* = 35 Å, *y* = 35 Å, and *z* = 35 Å. This translates into the smallest distance of about 14 Å between the adjacent periodic images of the water droplet. Apparently, the size of the box is large enough to neglect any interactions between the adjacent periodic images of the water droplet. Prior to the BOMD simulations, the system was fully relaxed using a DFT method, in which the exchange and correlation interaction is treated with the Becke–Lee–Yang–Parr (BLYP)^26^ functional. The Grimme's dispersion correction method^27^ is applied to account for the weak dispersion interactions. A double-ζ Gaussian basis set combined with an auxiliary basis set^28^ and the Goedecker–Teter–Hutter (GTH) norm-conserved pseudopotentials^29^ are adopted to treat the valence and the core electrons, respectively. An energy cutoff of 280 Rydberg is set for the plane wave basis set and 40 Rydberg for the Gaussian basis set. The BOMD simulations were carried out in the constant volume and temperature ensemble, with the Nose–Hoover chain method for controlling the temperature (300 K) of the system. The integration step is set as 1 fs, which has been previously shown to achieve sufficient energy conservation for the water system.^22b–d^

In addition, the gas-phase reaction of CH_2_OO with H_2_S and H_2_S–H_2_O complex was examined. The geometries of all the stationary points on the potential energy surfaces for both reactions were fully optimized using the BLYP^26^ functional, and aug-cc-pVTZ^30^ basis set. The harmonic vibrational frequency analyses were performed to ascertain the identity of the stable minima and transition states, as well as for estimating the zero-point electronic energy corrections for the reactants, products, transition states, and intermediates. The single point calculations using the coupled cluster single and double substitution method with a perturbative treatment of triple excitations [CCSD(T)]^31^ and the aug-cc-pVTZ basis set were performed on the BLYP optimized geometries to further improve the energetics. These DFT and CCSD(T) calculations were carried out using Gaussian09 ^32^ software.

## Results and discussion

### Gas-phase reaction

**Fig. 2:**
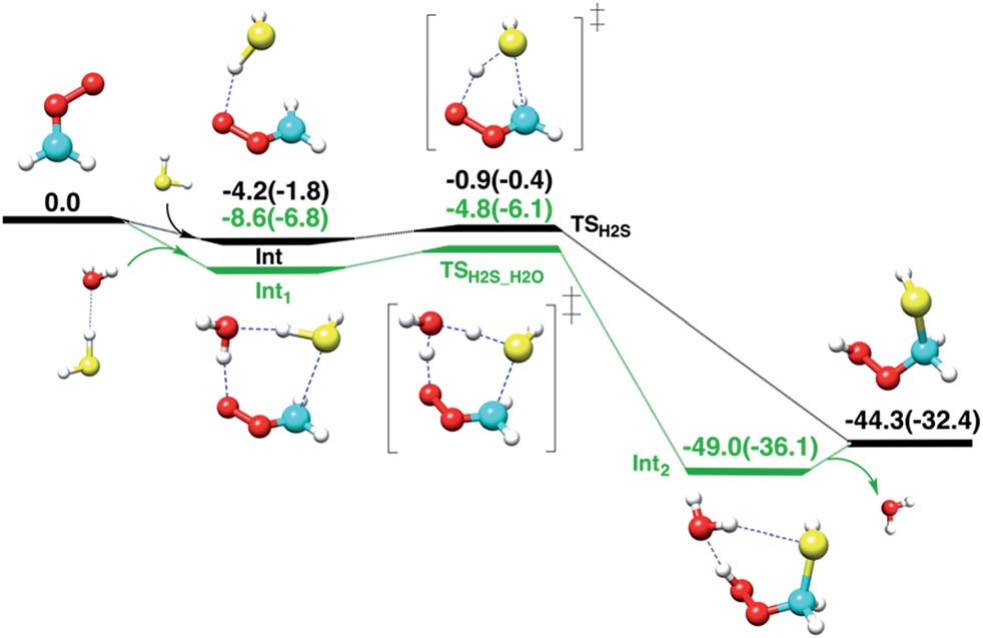
The CCSD(T)/aug-cc-pVTZ//BLYP/aug-cc-pVTZ computed reaction profiles in units of kcal mol^–1^ (at 298.15 K, 1 atm) for the reactions of CH_2_OO with hydrogen sulfide and hydrogen sulfide–water complex. The white, red, gray and yellow spheres represent H, O, C and S atoms, respectively. The BLYP/aug-cc-pVTZ calculated values are given in parentheses.

First, the gas-phase reaction between CH_2_OO and H_2_S with or without one H_2_O molecule is examined at the CCSD(T)/aug-cc-pVTZ//BLYP/aug-cc-pVTZ level of theory. Although the Criegee intermediate entails appreciable multiconfigurational character,^33^ especially when the substituents on the Criegee moiety are electron-withdrawing groups,^34^ previous theoretical studies^34–37^ have shown that the coupled-cluster//DFT level of theory can provide reasonable description of its unimolecular and bimolecular chemistry due in part to relatively small fraction (<15%) of the total wave-function that accounts for multi-reference nature of Criegee intermediate. The computed reaction profiles for the gas-phase CH_2_OO + H_2_S and CH_2_OO + H_2_S···H_2_O reactions are shown in Fig. 2. The uncatalyzed addition of H_2_S across the –COO moiety of CH_2_OO occurs in a concerted manner, which leads to the exothermic formation of (HS)CH_2_(OOH). The computed barrier for the uncatalyzed reaction, with respect to the CH_2_OO···H_2_S complex, is 3.3 kcal mol^–1^ at the CCSD(T)/aug-cc-pVTZ//BLYP/aug-cc-pVTZ level. This barrier is 1.9 kcal mol^–1^ larger than that calculated at the BLYP/aug-cc-pVTZ level. The CCSD(T)/aug-cc-pVTZ//BLYP/aug-cc-pVTZ calculated exothermicity of the reaction is 44.3 kcal mol^–1^, 11.9 kcal mol^–1^ larger than that calculated with BLYP/aug-cc-pVTZ method. These comparisons suggest that the electron correlation appreciably impacts the reaction barrier and energetics, and must be accounted for. For the concerted CH_2_OO + H_2_S···H_2_O reaction, the transition state is submerged below free reactants, and the product complex (HS)CH_2_(OOH)···H_2_O is 4.7 kcal mol^–1^ more stable than separated (HS)CH_2_(OOH) and H_2_O at the CCSD(T)/aug-cc-pVTZ//BLYP/aug-cc-pVTZ level. Notably, the calculated barrier for the CH_2_OO + H_2_S···H_2_O reaction relative to the Int_1_, is 3.8 kcal mol^–1^, 0.5 kcal mol^–1^ larger than that for the uncatalyzed reaction.

### Air/water interface reaction

There is literature precedence suggesting that aerosols, fog and cloud water may play a key role in the atmospheric chemistry.^22^ Here, we provide the BOMD simulation evidence of a reaction between the Criegee intermediate, CH_2_OO, and H_2_S on the air/water surface. Unlike in the gas phase, the CH_2_OO···H_2_S reaction at the air/water interface can be observed directly in the BOMD simulation trajectory after a few ps, and the reaction follows both concerted and stepwise mechanisms. We have performed total 16 BOMD simulations to study the CH_2_OO···H_2_S reaction on the water droplet, one BOMD simulation to study the dynamic behaviour of CH_2_OO on the water droplet, and one BOMD simulation to study the dynamic behaviour of H_2_S on the water droplet. In 8 of the 16 simulations, the reaction between CH_2_OO and H_2_O molecules has been observed whereas in another 8 simulations, the CH_2_OO···H_2_S reaction occurs. It is important to note that the CH_2_OO···H_2_S reaction on the water droplet is only observed when CH_2_OO and H_2_S are placed no farther than 4.0 Å. Since the CH_2_OO···H_2_O reaction at the air/water interface has been described in detail elsewhere,^22*b*^ here, we mainly focus on the CH_2_OO···H_2_S reaction. The most dominant mechanistic pathway for the CH_2_OO···H_2_S reaction involves the single water molecule-mediated concerted reaction, which accounts for 50% of the total number of CH_2_OO···H_2_S reactions on the water droplet. A significant fraction (25%) of the CH_2_OO···H_2_S reactions involve the concerted reaction mediated by two H_2_O molecules. All the possible reaction pathways for the CH_2_OO···H_2_S reaction on the water droplet are described in detail below:

### Concerted reaction at the air/water interface

**Fig. 3:**
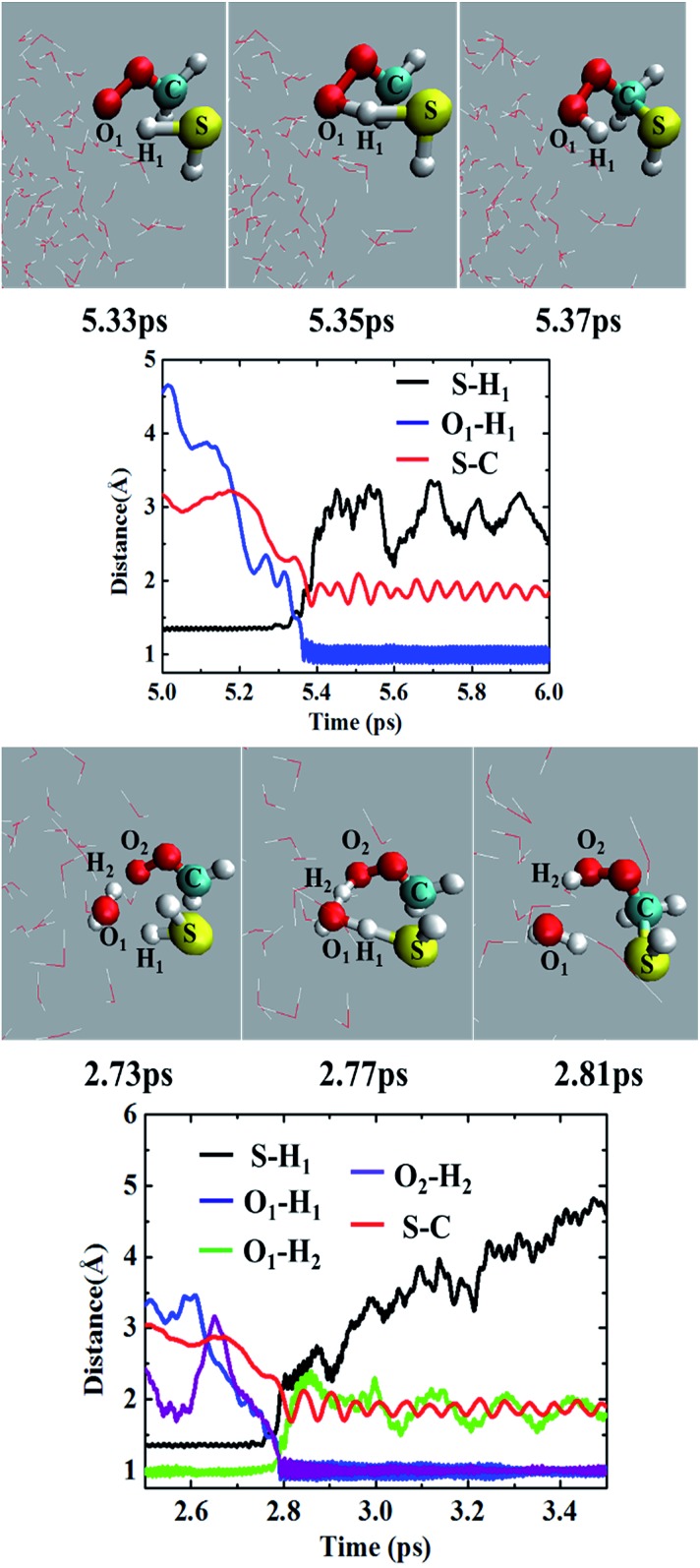
Top two panels: (upper panel) snapshot structures taken from the BOMD simulation of the reaction between CH_2_OO and H_2_S, which illustrates the concerted formation of the (HS)CH_2_(OOH) without the involvement of any water molecule in the droplet (cloud model), and (lower panel) time evolution of key bond distances, S–H_1_, C–S and O_1_–H_1_, in the course of BOMD simulation. Lower two panels: (upper panel) snapshot structures taken from the BOMD simulation for the single water molecule-mediated concerted reaction between CH_2_OO and H_2_S, and (lower panel) time evolution of key bond distances, S–H_1_, C–S, O_1_–H_1_, O_1_–H_2_ and O_2_–H_2_.

Most of the CH_2_OO···H_2_S reaction (87.5%) occurs concertedly. There are three different concerted pathways that have been observed in the BOMD simulations: (i) the direct reaction between CH_2_OO and H_2_S (12.5%), (ii) one H_2_O molecule-mediated reaction (50%), and (iii) two H_2_O molecules-mediated reaction (25%). The mechanistic details of the concerted direct reaction are quite similar to that of the uncatalyzed reaction in the gas phase. As shown in Fig. 3, the formation of a prereaction complex between CH_2_OO and H_2_S is observed at ∼5.33 ps, where the C–S bond is 3.1 Å long, and there is virtually no interaction between the terminal Criegee oxygen and thiol proton (S–H_1_). At ∼5.35 ps, the CH_2_OO···H_2_S complex looks more like the transition state in the gas phase, where the C–S bond is shortened to 1.9 Å while the S–H_1_ bond is elongated to 1.8 Å. There is also a 1.6 Å bond between the terminal Criegee oxygen and thiol proton (O_1_–H_1_). This complex converts into the final product, (HS)CH_2_(OOH) (Movie S1†) at ∼5.37 ps. The time evolution of key bond distances clearly supports the formation of (HS)CH_2_(OOH). In a recent study,^22*b*^ a sizable fraction of the CH_2_OO···H_2_O reactions at the air/water interface has been shown to follow a similar concerted mechanism. The gas-phase calculations suggest that (HS)CH_2_(OOH) is formed with an excess energy of 44.3–44.7 kcal mol^–1^. At the air/water interface, this excess energy will be absorbed by the water droplet and the surrounding environment (due to the enforcement of the constant-temperature condition) and (HS)CH_2_(OOH) will remain bound to the water surface *via* hydrogen bonding interaction. Alternatively, (HS)CH_2_(OOH) might decompose into HCHS and H_2_O_2_. This reaction is a proton transfer event and might also be mediated by the interfacial water molecules. However, this reaction in the gas-phase involves a barrier of over 49.0 kcal mol^–1^, and is, thus, unlikely to be observed *via* BOMD simulations.

In the BOMD simulations, the one H_2_O molecule-mediated concerted CH_2_OO···H_2_S reaction is the most dominant pathway (Fig. 3 and Movie S2†). The reaction involves the formation of a pre-reaction complex involving CH_2_OO, H_2_S and one H_2_O molecule at 2.73 ps. At this point, the C–S bond is 3.0 Å long, and H_2_S is hydrogen-bonded to H_2_O (O_1_–H_1_ = 2.2 Å), which, in turn, is hydrogen-bonded to the terminal oxygen of CH_2_OO (O_2_–H_2_ = 2.2 Å). At 2.77 ps, the cyclic CH_2_OO···H_2_S···H_2_O complex transforms into a transition state like intermediate in which the S–C bond is 2.4 Å long, the S–H_1_ bond is 1.6 Å long whereas the O_1_–H_1_, O_1_–H_2_, and O_2_–H_2_ bonds are nearly 1.4 Å long. This intermediate immediately converts into the water-bound final product, (HS)CH_2_(OOH)···H_2_O at 2.81 ps. The time evolution of key bond distances, S–C, S–H_1_, O_1_–H_1_, O_1_–H_2_, and O_2_–H_2_ corroborate the formation of the postreaction complex in this concerted pathway. The BOMD simulations also suggest the concerted reaction mediated by two H_2_O molecules. The reaction occurs on the ps time scale and is mediated by the prereaction and postreaction complexes, respectively. The formation of the hydrogen-bonded (HS)CH_2_(OOH) in this pathway is also supported by the time evolution profiles of key bond distances (Fig. S1 and Movie S3†).

### Stepwise reaction at the air/water interface

**Fig. 4:**
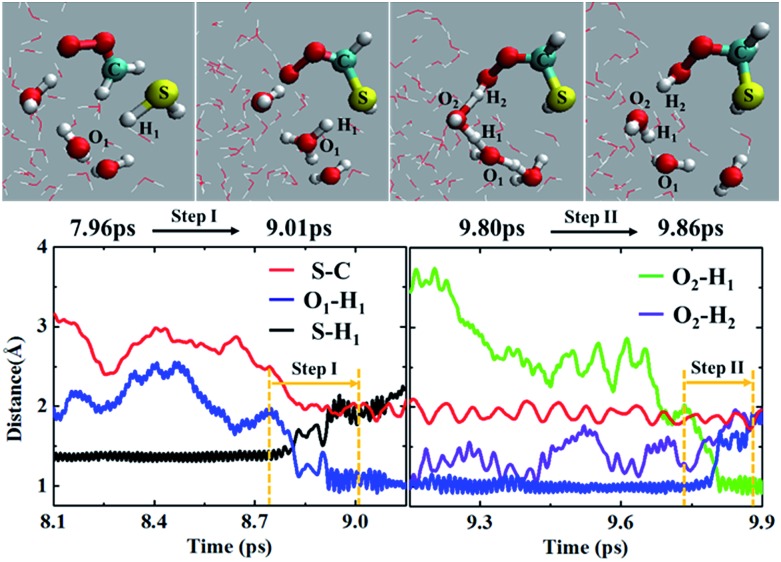
(Top panel) Snapshot structures taken from the BOMD simulation of the reaction between CH_2_OO and H_2_S, which illustrates the formation of the (HS)CH_2_(OOH) *via* a stepwise mechanism. (Lower panel) Time evolution of key bond distances, S–H_1_, S–C, O_1_–H_1_, O_2_–H_1_ and O_2_–H_2_, involved in the stepwise reaction.

In addition to the concerted mechanism, a noticeable fraction of the CH_2_OO···H_2_S reaction at the air/water interface (12.5%) also proceeds in a stepwise manner. In the first step, the breakage of H_1_–S bond of H_2_S occurs, leading to the concerted formation of C–S bond and (H_3_O)^+^. This event occurs at ∼9.01 ps. The time evolution profiles of C–S, S–H_1_ and O_1_–H_1_ bond distances (Fig. 4 and Movie S4†) clearly show the formation of (HS)CH_2_(OO)^–^ in the first step. At this point, the S–H_1_ bond is elongated to ∼2.0 Å whereas the O_1_–H_1_ bond is more like a normal O–H bond (∼1.0 Å). These structural changes imply the breakage of S–H_1_ bond, and the formation of C–S bond and (H_3_O)^+^, respectively. Notably, there exists no interaction between the terminal Criegee oxygen and hydrogen of nearby H_2_O molecule at this stage (O_2_–H_2_ ∼ 1.0 Å). In the second step, one water molecule, which is hydrogen-bonded to (H_3_O)^+^, catalyses the proton H_2_ transfer from (H_3_O)^+^ to the terminal Criegee oxygen. Following a structural reorganization, a ring-like structure including (H_3_O)^+^, two H_2_O molecules, and the terminal oxygen of (HS)CH_2_(OO)^–^ is formed at ∼9.80 ps. This configuration looks like a transition state, showing the two concerted proton transfers, (H_3_O)^+^ → H_2_O → (HS)CH_2_(OO)^–^. In this configuration, both the O_1_–H_1_ and O_2_–H_2_ bonds are elongated to ∼1.5 Å, suggesting the bond formation between the terminal Criegee oxygen and the hydrogen of a nearby water molecule, H_2_. The formation of (HS)CH_2_(OOH) is complete at ∼9.86 ps. The time insensitivity of C–S bond distance beyond 9.0 ps also supports the proton transfer from the water droplet to (HS)CH_2_(OO)^–^ in the second step.

Recently, the CH_2_OO···H_2_O reaction at the air/water interface has been found to follow the stepwise mechanism, in addition to the well-known concerted one.^22*b*^ It is interesting to compare the stepwise mechanism for the CH_2_OO···H_2_S reaction with that for the analogous CH_2_OO···H_2_O reaction. The major difference between the two stepwise mechanisms is that for the CH_2_OO···H_2_O reaction, the second step proton transfer from (H_3_O)^+^ to the terminal Criegee oxygen of (HO)CH_2_(OO)^–^ occurs without the involvement of any additional H_2_O molecule, whereas for the CH_2_OO···H_2_S reaction, the proton transfer from (H_3_O)^+^ to the (HS)CH_2_(OO)^–^ is catalyzed by a H_2_O molecule.

The above analysis shows that the air/water interface can mediate the CH_2_OO···H_2_S reaction. To fully understand the role of water droplet, the hydration structure, orientation and dynamical behaviour of CH_2_OO and H_2_S on the water droplet is analysed next.

### Effects of water droplet

**Fig. 5:**
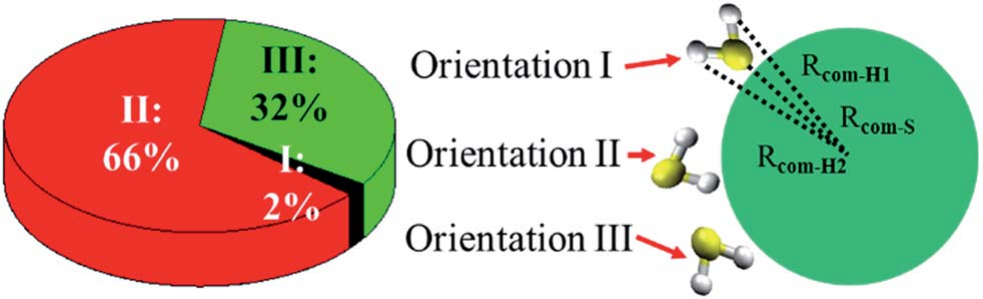
Orientations (I, II, and III) of H_2_S at the air/water interface and their probabilities. Orientation I corresponds to *R*_com-S_ < *R*_com-H1_ < *R*_com-H2_. Orientation II corresponds to *R*_com-H1_ < *R*_com-H2_ < *R*_com-S_. Orientation III corresponds to *R*_com-H1_ < *R*_com-S_ < *R*_com-H2_. *R*_com-H1_, *R*_com-H2_ and *R*_com-S_ represent the distance between S (H_2_S), H1 (H_2_S), and H2 (H_2_S) and center of mass (COM) of the water droplet. H1 is the hydrogen atom in H_2_S with shorter distance from the COM of the water droplet.

Fig. 5 shows three typical orientations (I, II, and III) of H_2_S on the water droplet and their corresponding probabilities. The orientation II has the highest probability, indicating the H atom of H_2_S prefers to interact with the water droplet due to hydrogen bonding H(H_2_S)···O(H_2_O) interaction. See Fig. S2† for the radial distribution function. Closer inspection of orientation II shows that only one of the two H atoms in H_2_S forms hydrogen bond with the water droplet (see Fig. S3†). This allows H_2_S to transfer one of its H atoms to H_2_O molecules easily, which, in turn, favours the CH_2_OO···H_2_S reaction. Similarly, the orientation III, where one H atom in H_2_S interacts with water, would favour the proton transfer from H_2_S to the water droplet.

**Fig. 6:**
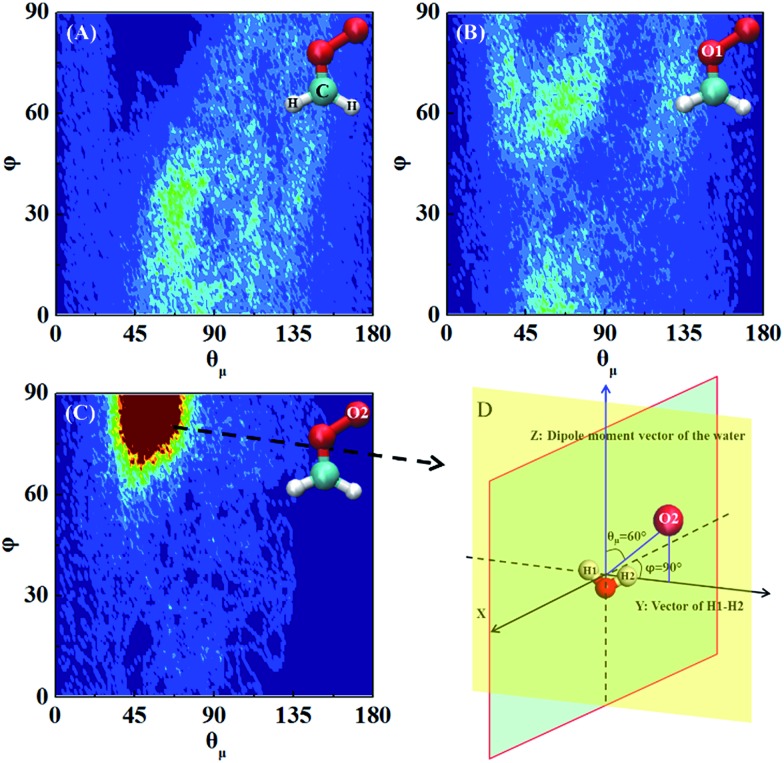
Joint probability distributions *P*(*θ*_*μ*_,*φ*) computed for water molecules within the first hydration shell of the CH_2_OO at air/water interface; (A) –CH_2_ (B) O1 (C) O2. Red and blue colours represent high and low probabilities, respectively. The high probability orientation between CH_2_OO and H_2_O is shown in (D).

Next, the hydration of CH_2_OO on the surface of the water droplet is investigated by computing the joint probability distributions (*P*(*θ*_*μ*_,*φ*)) for H_2_O molecules within the first hydration shell of CH_2_OO (Fig. 6). *P*(*θ*_*μ*_,*φ*) is uniquely defined by two angular coordinates, *θ*_*μ*_ and *φ*. The *θ*_*μ*_ is the angle formed by the vector between the H_2_O oxygen and the specified atoms *r⇀*_OS_, and the dipole moment vector of the H_2_O molecule *μ⇀*, where *r⇀*_OS_ points to the direction of the specified atoms. The coordinate *φ* is the angle made by the projection of *r⇀*_OS_ onto a local *XY*-plane and the local *X*-axis, which is normal to the H–O–H plane. Based on the computed *P*(*θ*_*μ*_,*φ*), it can be seen that there is no obvious strong interaction between the water droplet and either –CH_2_ or O1 of CH_2_OO. However, the O_2_ atom of CH_2_OO interacts strongly with a nearby H_2_O molecule (Fig. 6C). See also Fig. S4† for the radial distribution function. In the high probability region (Fig. 6D), the *φ* is around 90° and *θ*_*μ*_ is close to 60°, which is indicative of the fact that there exists a strong hydrogen bond, O2(CH_2_OO)···H(H_2_O), and the H atoms of nearby H_2_O in the droplet are easily transferrable to CH_2_OO.

### “Trapping” effects of the water droplet

Why does the concerted CH_2_OO–H_2_S reaction without the direct involvement of nearby water molecules on the water droplet occur on a ps time scale? According to the hydration structure analysis, the H atom of H_2_S and O_2_ atom of CH_2_OO prefer to interact with H_2_O near the surface of the droplet whereas, the S atom of H_2_S and C atom of CH_2_OO exhibit relatively weak interaction with the water droplet, and both are located at a further distance from the droplet, which implies the possibility of C–S bond forming reaction. The H_2_O molecules in the droplet can make the configurations of H_2_S and CH_2_OO favourable to form pre-reaction complex, and stabilize such configurations due to trapping effect, thereby allowing the concerted reaction to occur within ps.

## Conclusions

In summary, we have shown evidence from the Born–Oppenheimer molecular dynamics simulations that at the air/water interface, a significant fraction of Criegee intermediate, CH_2_OO (50%) reacts with H_2_S. Importantly, the reaction of CH_2_OO with H_2_S at the air/water surface occurs on a picosecond time scale and follows both concerted and stepwise mechanisms with former being the dominant pathway. The concerted reaction between CH_2_OO and H_2_S mediated by one and two water molecules on the water droplet are two of the most dominant pathways. An important mechanistic difference between the CH_2_OO···H_2_S and CH_2_OO···H_2_O reactions is that the proton transfer in the stepwise H_2_S reaction is mediated by a water molecule, whereas the proton transfer in the stepwise H_2_O reaction occurs without the involvement of any additional water molecule. To our knowledge, this is the first simulation evidence of C–S bond formation *via* Criegee···H_2_S reaction at the air/water interface. This new Criegee···H_2_S chemistry could be a new oxidation pathway for H_2_S in terrestrial, geothermal and volcanic regions.

## References

[cit1] Evans N. J., Townes C. H., Weaver H. F., Williams D. R. (1970). Science.

[cit2] Agundez M., Fonfria J. P., Cernicharo J., Pardo J. R., Guelin M. (2008). Astron. Astrophys..

[cit3] Okazaki R., in Organosulfur Chemistry, ed. P. D. Page, Academic Press, London, 1995, p. 226.

[cit4] Vedejs E., Perry D. A., Houk K. N., Rondan N. G. (1983). J. Am. Chem. Soc..

[cit5] McGregor W. M., Sherrington D. C. (1993). Chem. Soc. Rev..

[cit6] Allen K. D., Miller D. V., Rauch B. J., Perona J. J., White R. H. (2015). Biochemistry.

[cit7] Kumar M., Francisco J. S. (2016). Chem.–Eur. J..

[cit8] Judge R. H., King G. W. (1975). Can. J. Phys..

[cit9] Kumar M., Francisco J. S. (2016). Angew. Chem., Int. Ed..

[cit10] Criegee R. (1975). Angew. Chem., Int. Ed..

[cit11] Welz O., Savee J. D., Osborn D. L., Vasu S. S., Percival C. J., Shallcross D. E., Taatjes C. A. (2012). Science.

[cit12] Osborn D. L., Taatjes C. A. (2015). Int. Rev. Phys. Chem..

[cit13] Leisch H., Morley K., Lau P. (2011). Chem. Rev..

[cit14] Van Ornum S. G., Champeau R. M., Pariza R. (2006). Chem. Rev..

[cit15] US EPA, 1993 Report to Congress on hydrogen sulfide air emis- sions associated with the extraction of oil and natural gas. Research Triangle Park, NC, US Environmental Protection Agency, Office of Air Quality Planning and Standards EPA/453/R93045; NTIS Publication No. PB941312240.

[cit16] Brimblecombe P., Air Composition and Chemistry, Cambridge University Presss, 1996.

[cit17] Bogner J. and Heguy D., Msw Management, March-April 2004.

[cit18] Lee S., Xu Q., Booth M., Townsend T. G., Chadik P., Bitton G. (2006). Waste Manag..

[cit19] Takemoto B. K., Noble R. D., Harrington H. M. (1986). New Phytol..

[cit20] Hill F. B. (1973). Brookhaven Symp. Biol..

[cit21] Vereecken L., Glowacki D. R., Pilling M. J. (2015). Chem. Rev..

[cit22] Ravishankara A. R. (1997). Science.

[cit23] Gultepe I., Isaac G. A. (2004). Q. J. R. Meteorol. Soc..

[cit24] Pouliquen F., Blanc C. and Arretz E., et al., Hydrogen sulfide, in Ullmann's encyclopedia of industrial chemistry, high-performance fibers to imidazole and derivatives, ed. B. Elvers, S. Hawkins and M. Revenscroft, VCH Publishers, Deerfield, Beach, FL, 1989, vol. A13, pp. 467–485.

[cit25] VandeVondele J., Krack M., Mohamed F., Parrinello M., Chassaing T., Hutter J. (2005). Comput. Phys. Commun..

[cit26] Becke A. D. (1988). Phys. Rev. A: At., Mol., Opt. Phys..

[cit27] Grimme S., Antony J., Ehrlich S., Krieg H. (2010). J. Chem. Phys..

[cit28] VandeVondele J., Hutter J. (2007). J. Chem. Phys..

[cit29] Hartwigsen C., Goedecker S., Hutter J. (1998). Phys. Rev. B: Condens. Matter Mater. Phys..

[cit30] Kendall R. A., Dunning Jr T. H., Harrison R. J. (1992). J. Chem. Phys..

[cit31] Noga J., Bartlett R. J. (1987). J. Chem. Phys..

[cit32] Frisch M. J., Trucks G. W., Schlegel H. B., Scuseria G. E., Robb M. A., Cheeseman J. R., Scalmani G., Barone V., Mennucci B., Petersson G. A., et al., Gaussian 09, Revision D.01, Gaussian, Inc., Wallingford CT, 2009.

[cit33] Kalinowski J., Rasanen M., Heinonen P., Kilpelainen I., Gerber R. B. (2014). Angew. Chem., Int. Ed..

[cit34] Anglada J. M., Gonzalez J., Torrent-Sucarrat M. (2011). Phys. Chem. Chem. Phys..

[cit35] Liu F., Fang Y., Kumar M., Thompson W. H., Lester M. I. (2015). Phys. Chem. Chem. Phys..

[cit36] Kumar M., Busch D. H., Subramaniam B., Thompson W. H. (2014). Phys. Chem. Chem. Phys..

[cit37] Zhong J., Kumar M., Zhu C. Q., Francisco J. S., Zeng X. C. (2017). Angew. Chem., Int. Ed..

