# Supplementary material for: Criegee intermediate-hydrogen sulfide chemistry at the air/water interface
Source: Chem Sci. 2017 May 16;8(8):5385–91. doi: 10.1039/c7sc01797a (PMC5609144; doi:10.1039/c7sc01797a)
Supplement: Supplementary file 1 [file SC-008-C7SC01797A-s001.pdf]

Electronic Supplementary Material (ESI) for Chemical Science.
This journal is © The Royal Society of Chemistry 2017

Criegee Intermediate-Hydrogen Sulfide Chemistry at the Air/Water Interface

Manoj Kumar[#a], Jie Zhong[#a], Joseph S. Francisco[*a], Xiao Cheng Zeng[*ab]

[a]Department of Chemistry, University of Nebraska-Lincoln, Lincoln, Nebraska, 68588, United States
[b]Beijing Advanced Innovation Center for Soft Matter Science and Engineering, Beijing University of Chemical Technology, Beijing 100029, China

Part I: Electronic Supplementary Figures

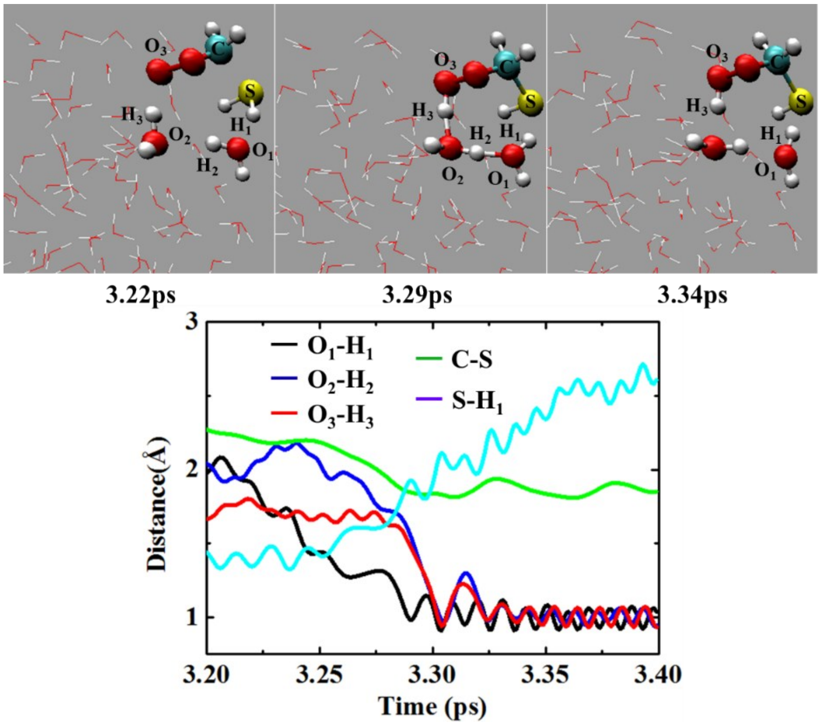

Figure S1. (Upper panel) snapshot structures taken from the BOMD simulation for the two water molecules-mediated concerted reaction between CH_2OO and H_2S, and (lower panel) time evolution of key bond distances, O_1-H_1, O_2-H_2, O_3-H_3, C-S and S-H_1.

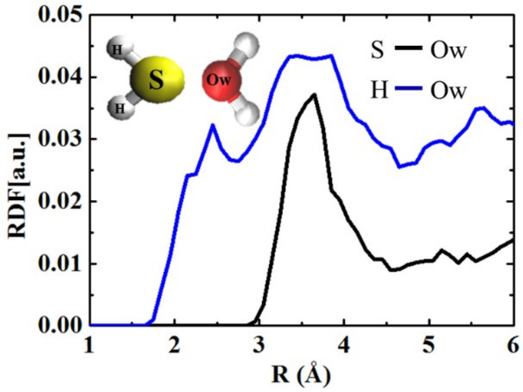

Figure S2. Radical distribution functions (in arbitrary unit) between H_2S and H_2O. Black and blue curves represent RDFs for $_{H2S}S\cdot\cdot O_{H2O}$, $_{H2S}H\cdot\cdot O_{H2O}$ in the surface region, respectively.

The site-dependent interaction between H_2S and water droplet is analyzed using the radial distribution function (RDF), as shown in Figure S2. The unit of RDF is arbitrary in the sense that it is a relative measurement of atom-site distribution *versus* the distance between two concerning atom sites. The RDF for H··Ow (H atom in H_2S, and O atoms in water droplet) shows the formation of the hydration layer around the H atom. The radius of the first hydration layer is around 2.2 Å, indicating the hydrogen bond formation of $_{H2S}H\cdot\cdot O_{H2O}$. The RDF for S··Ow (S atom in H_2S, and O atoms in water droplet) shows the hydration layer with a larger radius (3.7 Å) being formed by the terminal S atoms in SO_2, indicating weak interaction of $_{H2S}S\cdot\cdot O_{H2O}$.

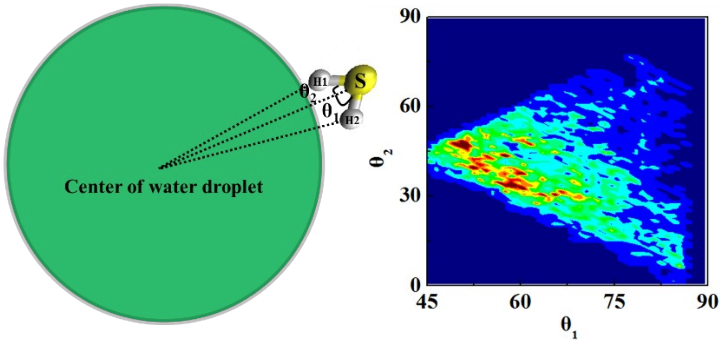

Figure S3. (Left) Definition of θ_1 and θ_2. Here, θ_1 is the angle between S···COM (center of mass of the water droplet) line and S···H1 line, where H1 is the H atom of H_2S with shorter distance to the center of mass of the water droplet. θ_2 is the angle between the S···COM line and S···H2 line. H2 is the H atom of H_2S with larger distance to the center of mass of the water droplet. (Right) The probability distributions P(θ_1, θ_2) computed for H_2S at the air-water interface.

The microstructure of orientation II is analysed in more detail. The angles of θ_1 and θ_2 as shown in the left panel of Figure S3 are defined to evaluate microscopic picture of orientation II, and their probability distribution is shown in the right panel of Figure S3. It can be seen that θ_1 is distributed over the angular region from 30° to 45°, while θ_2 is distributed over the angular region from 50° to 65°. This result indicates that only H1 atom in H_2S tends to form hydrogen bond with water droplet.

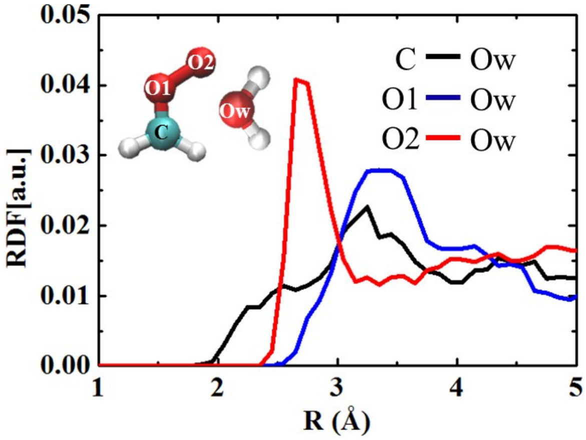

Figure S4. Radical distribution functions (in arbitrary unit) between CH_2OO and H_2O. Black, blue and red curves represent RDFs for $_{CH2OO}C\cdot\cdot O_{H2O}$, $_{CH2OO}O1\cdot\cdot O_{H2O}$, $_{CH2OO}O2\cdot\cdot O_{H2O}$ in the surface region, respectively.

The site-dependent interaction between CH_2OO and water droplet is analyzed using the radial distribution function (RDF), as shown in Figure S4. The RDF for O2··Ow (terminal O atom in CH_2OO, and O atoms in water droplet) shows a hydration layer with a smaller radius and a higher peak than that of either O1··Ow (middle O atom in CH_2OO, and O atoms in water droplet) or C··Ow (C atom in CH_2OO, and O atoms in water droplet), indicating that the dominant interaction between CH_2OO and water droplet stems from $_{CH2OO}O2\cdot\cdot O_{H2O}$.

Part II: BLYP/aug-cc-pVTZ calculated geometries of key species involved in the gas-phase reactions of CH_2OO with H_2S and H_2S-H_2O.

H_2S

S	0.000000	0.000000	0.104404
H	0.000000	0.975766	-0.835231
H	0.000000	-0.975766	-0.835231

H_2O

O	0.000000	0.119001	0.000000
H	0.768446	-0.476026	0.000000
H	-0.768446	-0.475978	0.000000

H_2S-H_2O

H	0.014190	0.023729	-0.001752
H	1.538980	1.255951	0.005096
H	-2.701070	-0.305318	0.769510
H	-2.706061	-0.295575	-0.770730
S	1.369524	-0.087714	-0.000340
O	-2.257303	0.090580	0.000415

CH_2OO

C	-1.087297	0.206269	-0.000009
H	-1.993584	-0.392577	-0.000084
O	1.196815	0.199456	0.000047
O	-0.001513	-0.466841	-0.000041
H	-1.045045	1.294042	0.000087

CH_2OO + H_2S Reaction

Int

C	-1.383536	1.069437	0.210456
O	-1.158970	-1.201937	0.196957
O	-1.639172	-0.033069	-0.376466
H	0.816292	-0.765543	0.039805
S	1.889776	0.076828	-0.119146
H	-1.758332	1.952716	-0.300660
H	-0.845244	1.072857	1.154415
H	2.237218	-0.025848	1.186113

TS

C	-0.886899	1.088037	0.235125
O	-1.160880	-1.152611	0.192585
O	-1.446043	0.115772	-0.391986
H	0.436323	-0.880325	0.077976
S	1.585198	-0.019812	-0.130264

H	-0.967101	2.054590	-0.259094
H	-0.596457	0.971991	1.273114
H	1.940846	-0.062779	1.176692

(HS)CH_2(OOH)

C	0.015654	0.835893	0.304873
O	-1.756308	-0.705406	0.185195
O	-1.187245	0.546219	-0.391210
H	1.237978	-1.183626	0.829425
H	-1.493386	-1.342825	-0.510845
S	1.473838	-0.242699	-0.115627
H	0.269332	1.845649	-0.036970
H	-0.140824	0.822120	1.387304

CH_2OO + H_2S-H_2O Reaction

Int_1

C	0.731138	-1.530932	0.239987
O	2.095730	0.308631	0.196243
O	1.536680	-0.820872	-0.443669
H	0.884572	1.563551	0.102567
H	0.233207	2.774794	-0.653505
H	-1.252776	0.825407	-0.096056
H	-2.392313	-0.160075	1.194409
S	-2.026213	-0.324897	-0.099890
H	0.344537	-2.408249	-0.270753
H	0.560784	-1.298633	1.287971
O	0.074410	2.148136	0.071636

TS

C	0.391934	-1.490901	0.130660
O	1.945534	0.189380	0.340810
O	1.313342	-0.833840	-0.481364
O	0.191648	2.006925	0.003352
H	1.002436	1.340712	0.119583
H	0.335099	2.493889	-0.826682
H	-0.885714	1.003564	-0.090681
H	-2.037472	-0.045755	1.267614
H	-0.003418	-2.333117	-0.432367
H	0.341467	-1.436889	1.214732
S	-1.794262	-0.186046	-0.058659

Int_2

C	1.150006	0.836659	-0.174941
O	-0.990326	1.325424	0.646678
O	-0.109533	1.330157	-0.555596
O	-2.157810	-1.073473	-0.275837

H	-2.610918	-1.724835	0.285521
H	0.953159	-1.022498	1.386814
H	-1.238039	-1.403068	-0.369164
H	-1.583334	0.565394	0.401606
S	1.270101	-1.029340	0.069675
H	1.799451	1.061532	-1.028195
H	1.519377	1.316098	0.736305
